# Biotechnological Approaches for Production of Artemisinin, an Anti-Malarial Drug from *Artemisia annua* L.

**DOI:** 10.3390/molecules27093040

**Published:** 2022-05-09

**Authors:** Jameel M. Al-Khayri, Wudali N. Sudheer, Vasantha V. Lakshmaiah, Epsita Mukherjee, Aatika Nizam, Muthu Thiruvengadam, Praveen Nagella, Fatima M. Alessa, Muneera Q. Al-Mssallem, Adel A. Rezk, Wael F. Shehata, Mahesh Attimarad

**Affiliations:** 1 Department of Plant Biotechnology, College of Agriculture and Food Sciences, King Faisal University, Al-Ahsa 31982, Saudi Arabia; arazk@kfu.edu.sa (A.A.R.); wshehata@kfu.edu.sa (W.F.S.); 2 Department of Life Sciences, CHRIST (Deemed to be University), Bangalore 560029, India; wudali.sudheer@res.christuniversity.in (W.N.S.); vasantha.vl@christuniversity.in (V.V.L.); 3 Amity Institute of Biotechnology, Amity University, Noida 201313, India; epsitamukherjee18@gmail.com; 4 Department of Chemistry, CHRIST (Deemed to be University), Bangalore 560029, India; aatika.nizam@christuniversity.in; 5 Department of Crop Science, College of Sanghuh Life Science, Konkuk University, Seoul 05029, Korea; muthu@konkuk.ac.kr; 6 Department of Food Science and Nutrition, College of Agriculture and Food Sciences, King Faisal University, Al-Ahsa 31982, Saudi Arabia; falissa@kfu.edu.sa (F.M.A.); mmssallem@kfu.edu.sa (M.Q.A.-M.); 7 Department of Pharmacology, College of Clinical Pharmacy, King Faisal University, Al-Ahsa 31982, Saudi Arabia; mattimarad@kfu.edu.sa

**Keywords:** *Artemisia annua*, Artemisinin, *Agrobacterium rhizogenes*, cell suspension culture, elicitation, bioreactor

## Abstract

Artemisinin is an anti-malarial sesquiterpene lactone derived from *Artemisia annua* L. (Asteraceae family). One of the most widely used modes of treatment for malaria is an artemisinin-based combination therapy. Artemisinin and its associated compounds have a variety of pharmacological qualities that have helped achieve economic prominence in recent years. So far, research on the biosynthesis of this bioactive metabolite has revealed that it is produced in glandular trichomes and that the genes responsible for its production must be overexpressed in order to meet demand. Using biotechnological applications such as tissue culture, genetic engineering, and bioreactor-based approaches would aid in the upregulation of artemisinin yield, which is needed for the future. The current review focuses on the tissue culture aspects of propagation of *A. annua* and production of artemisinin from *A. annua* L. cell and organ cultures. The review also focuses on elicitation strategies in cell and organ cultures, as well as artemisinin biosynthesis and metabolic engineering of biosynthetic genes in *Artemisia* and plant model systems.

## 1. Introduction

Medicinal plants have long been a component of human civilization. Plants are utilized to heal ailments and have saved people from countless pandemics, according to Ayurvedic writings and kindred scriptures. Medicinal plants are high in pharmacologically significant bioactive chemicals that define the plant’s medicinal properties. Artemisinin is one such medicinally important metabolite derived principally from the aerial parts of the medicinal herb *Artemisia annua* (L.). Artemisinin is a sesquiterpene lactone molecule with an endoperoxide structure that has anti-malarial properties [1]. *A. annua* is a member of the Asteraceae family that grows in tropical and subtropical climates. The plant is native to China and it is known as qinghao, commonly called sweet annie and worm wood in English. The plant is recorded in ancient Chinese texts as being used to cure fevers [2]. Studies revealed that artemisinin has significant potential in restricting the growth of pathogens in the body by accumulating in the digestive vacuole and ultimately alkylating the haem. Artemisinin-based combination therapy gained importance in the treatment of malaria along with chloroquine and sulphadoxine-pyrimethamine medicines [3]. According to the 2015 World Malaria Report, malaria is a disease of global health relevance, with 600,000 fatalities and the risk of death for 3.3 billion people [4]. *Plasmodium falciparum* is the causative organism for malarial infection, which completes its life cycle through multiple hosts [5].

Artemisinin was discovered by Youyou Tu in 1972, for which she was awarded the Nobel prize in 2015. Her extensive studies revealed that artemisinin is a colorless crystalline compound with the molecular formula C_15_H_22_O_5_ (282 Da) and a melting point of 156–157 °C [6]. Biosynthetically, artemisinin is majorly produced only in varieties of *A. annua* with glandular trichomes. Glandular trichomes in *Artemisia* are rich in transcriptional factors, which activate the genes responsible for the biosynthesis of artemisinin [7]. Despite the fact that artemisinin is an anti-malarial drug, it also shows a wide range of pharmacological activities. Studies reveal that artemisinin shows anti-cancer properties by blocking the cell cycles and also inhibits tumor angiogenesis [8]. Anti-inflammatory action is demonstrated by artemisinin and its related compounds/derivatives. It has been observed that these pharmacologically bioactive compounds lower serum antibody levels as well as the relevant cytokines [9]. The anti-microbial activity [10] and anti-viral activity [11] of artemisinin and its derivatives are studied, suggesting that it is one of the significant bioactive molecules with multiple pharmacological activities.

As there are so many possible pharmacological aspects, there is a need for artemisinin production and a rise in its output. Conventional artemisinin production will not be enough to fulfill demand. As a result, current biotechnological approaches such as plant cells, tissue and organ cultures, bioreactor-based scaling up, and metabolic engineering strategies must be utilized. The present review focuses on the significance of artemisinin, its biosynthesis, production using in vitro technologies, such as tissue and organ cultures, elicitation strategies for boosting the yield, and bioreactor-based scaling up of production of artemisinin. This review also helps in understanding the metabolic engineering element, which allows us to understand the biosynthesis and genes involved in artemisinin production, ultimately allowing us to build a better strategy for artemisinin production.

## 2. Regeneration Studies

The in vitro regeneration approach allows us to conserve and proliferate rare and therapeutic plant species for the extraction of diverse bioactive compounds. Since *A. annua* is a medicinal herb with significant anti-malarial effects, it is critical to understand how to regenerate it, utilizing in vitro techniques to boost its bioactive compound content while also preserving its excellent quality germplasm.

### 2.1. Direct Organogenesis

Direct organogenesis is associated with the formation of organs such as shoots and roots directly from cultured explants, avoiding the callusing phase of in vitro regeneration. Seeds, leaves, and shoot regions are some of the successful explants used for direct organogenesis in *A. annua*.

Multiple shoots were developed from seed explants collected from the European region when inoculated on an MS medium supplemented with 0.1 mg/L of benzyl adenine (BA) along with 1.0 mg/L of indole-3-acetic acid (IAA), and subsequently, when the shoots were sub-cultured on a Murashige and Skoog (MS) medium supplemented with 1.0 mg/L naphthalene acetic acid (NAA) along with 0.1 mg/L kinetin (KN), gave roots [12]. Optimal shoot length, root length, and the number of nodes were observed when seed explants were inoculated on MS media fortified with 0.1 mg/L KN along with 0.01 mg/L NAA. Similarly, media fortified with 0.1 mg/L BA along with 0.01 mg/L of NAA showed callus induction [13]. Surface sterilized seeds, when inoculated on an MS medium supplemented with 4.4 µM BA along with 0.35 µM NAA, showed good induction capacity; optimal multiplication of shoots was observed on an MS medium supplemented with 0.9 µM BA and 0.05 µM of NAA [14].

Induction of multiple shoots was observed with 100% efficiency (57 shoots per explant) when stem explants were inoculated on an MS medium supplemented with 0.1 mg/L thidiazuron (TDZ) and successive rooting was established on an MS basal medium [15]. Nodal explants, when inoculated on an MS medium supplemented with 0.2 mg/L benzyl amino purine (BAP) in combination with 0.2 mg/L NAA, showed the highest shoot multiplication capacity [16]. The MS medium supplemented with 4.44 µM BAP showed optimal induction capacity of shoots when nodal explants were inoculated. These shoots were elongated when sub-cultured on an MS medium supplemented with 1.44 µM gibberellic acid (GA_3_) along with 10% coconut milk. These developed shoots were subjected to rooting when further transferred on a half-strength MS medium supplemented with indole-3-butyric acid (IBA) at 2.46 µM concentration [17]. Adventitious multiple shoots were developed from nodal explants when inoculated on an MS medium supplemented with 10 µM 2-iP and rooted on a medium supplemented with 5.0 µM NAA [18]. The MS medium supplemented with 0.8 mg/L BAP combined with 0.1 mg/L IBA showed optimal shoot induction (98.75 ± 2.50) and the highest multiplication capacity was recorded when nodal explants were inoculated on an MS medium supplemented with 1.0 mg/L BAP along with 0.1 mg/L IBA (8.05 ± 0.66 per explant). The best rooting with ideal root length and number was observed on a half-strength MS medium fortified with 0.5 mg/L IBA [19].

Explants, especially leaf and petiole obtained from seedlings, showed 100% regeneration efficiency when compared to greenhouse-grown plants. It is seen that leaves and petiole explants when inoculated on an MS medium supplemented with 1.0 mg/L TDZ showed direct organogenesis with shooting and rooting [20]; especially, leaf explants, when inoculated on MS media along with 1.0 mg/L BAP and 0.05 mg/L NAA, induced shoots. However, the addition of silver nitrate at a 2.0 mg/L concentration helped in a significantly higher regeneration capacity [21]. MS media supplemented with 0.5 mg/L NAA in combination with 2.0 mg/L BA showed the highest frequency in shooting, and roots were developed when sub-cultured on a 0.1 mg/L IBA medium [22].

The nodal segments from the inflorescence of *A. annua* developed multiple shoots when inoculated on an MS medium supplemented with 1.0 mg/L BAP. Subsequent subculturing of shoots on MS media incorporated with 2.0 mg/L IBA developed roots [23]. Table 1 depicts the data of comprehensive research on in vitro regeneration of *A. annua* using various concentrations and combinations of auxins and cytokinins, as well as their responses.

### 2.2. Indirect Organogenesis

Indirect organogenesis refers to the formation of organs such as shoots and roots following the intrusion of the callusing stage. Callusing is a very important stage, which helps in the establishment of cell suspension cultures, usually used for metabolite production. Multiple shoots were developed from calluses induced on media supplemented with 5.4 µM NAA upon further sub-culturing on an MS medium supplemented with 13.32 µM BA and 1.08 µM of NAA [24]. Hypocotyl explants, when inoculated on an MS medium supplemented with a combination of 0.5 µM NAA along with 13.0 µM BAP and 0.3 µM GA_3_, initiated the growth of calluses, and further sub-culturing on the same medium developed multiple shoots [25].

Leaf explants of *A. annua*, when inoculated on an MS medium supplemented with 0.1 mg/L BAP along with 0.05 mg/L NAA, helped in the induction of friable calluses. Further sub-culturing on an MS medium supplemented with 0.4 mg/L BAP along with 0.2 mg/L NAA exhibited multiple shoot regeneration [16]. Leaf explants, when inoculated on an MS medium supplemented with 1.0 mg/L BAP along with 0.05 mg/L NAA, induced calluses, and further organogenesis was established [26]. The MS medium supplemented with 0.5 mg/L NAA or 2,4-D along with 0.5 mg/L BAP showed callusing, and upon sub-culturing on media supplemented with 0.25 mg/L NAA in combination with 1.0 mg/L BAP, helped in optimal shooting and rooting. It was observed in 0.5 MS supplemented with 0.1 mg/L IBA [27]. Table 2 depicts the data of comprehensive research on in vitro regeneration (in-direct organogenesis) of *A. annua* using various concentrations and combinations of auxins and cytokinins, as well as their responses.

### 2.3. Somatic Embryogenesis

Employing somatic tissues or cells for the development of the whole plant is the chief characteristic of somatic embryogenesis. Studies suggest that somatic embryogenesis is one of the most successful techniques which helps us to better understand the development of plants and is regarded as one of the best approaches to clonal propagation and disease-free varieties of plants [28]. Various embryos like globular, torpedo, heart and cotyledon embryos were developed from leaf explants when inoculated on an MS medium supplemented with 0.6 mg/L TDZ in combination with 0.1 mg/L IBA. Further rooting of the adventitious buds was seen on the MS medium fortified with 0.5 mg/L NAA [26].

## 3. Biosynthesis of Artemisinin from *Artemisia annua* L.

**Figure 1:**
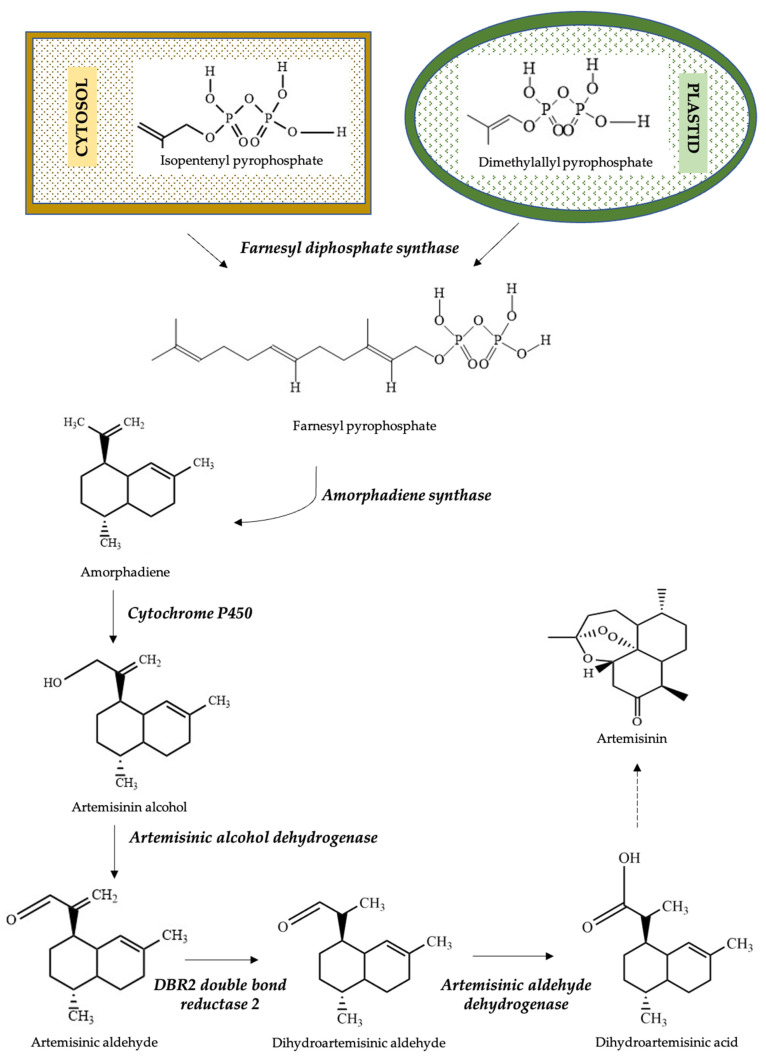
Biosynthetic pathway for the production of Artemisinin from *Artemisia annua*.

The biosynthetic pathway of artemisinin in *A. annua* has not been explored completely, but a basic understanding has been established (Figure 1). It is seen that precursors for the artemisinin biosynthesis are produced from mevalonate (MVA) and non-mevalonate pathways (MEP) [29]. Isopentenyl pyrophosphate (IPP) and dimethylallyl pyrophosphate (DMAPP) from MVA and MEP pathways regulated by 3-hydroxy-3-methylglutaryl-CoA reductase (HMGR), 1-deoxyxylulose 5-phosphate synthase (DXS), or 1-deoxyxylulouse 5-phosphate reductor isomerase (DXR) results in the formation of farnesyl diphosphate (FPP) through farnesyl diphosphate synthase (FPS). Later, amorpha-4, 11-diene was produced with the help of the enzyme amorphadiene synthase (ADS) from farnesyl diphosphate by cyclization. Further hydroxylation of amorpha-4,11-diene by cytochrome P450 (CYP71AV1) leads to the formation of artemisinic alcohol. Keat et al. (2006) proved that cDNAs isolated and coded for CYP71AV1 were cloned in the *Saccharomyces cerevisiae* system resulting in the formation of intermediates like alcohols and aldehydes, which later form artemisinin [30]. Oxidation of artemisinic alcohol with the help of dehydrogenases formed artemisinic aldehyde [31]. Artemisinic aldehyde later, with the help of DBR2 (Double bond reductase 2), formed dihydroartemisinic aldehyde. Later, aldehyde dehydrogenases, like Aldh1, form artemisinin by forming various intermediates, such as dihydroartemisinic acid. Studies revealed that all the enzymes such as ADS, CYP71AV1, DBR2, and ALDH1 were discovered in the glandular trichomes of the *Artemisia* plant [32]. In biosynthetic production, artemisinin is produced from dihydroartemisinic acid through various non-enzymatic reactions, in which molecular oxygen and light are required for the formation of ketoenol. The formed ketoenol forms hydroperoxide upon reacting with ground state oxygen and further results in the formation of artemisinin [33]. Xie et al., 2016, characterized the reactions involved in the biosynthesis of artemisinin, i.e., cyclization, oxidoreduction, alcohol dehydrogenation, aldehyde dehydrogenation, and trioxane formation [34].

## 4. In Vitro Production of Artemisinin from Artemisia

In vitro technology has been used to produce high-quality plant secondary metabolites with pharmaceutical applications and the highest commercial value. When compared to conventional methods of production, in vitro generation of bioactive metabolites from plants is one of the most successful solutions for long-term sustainability. The various means of producing metabolites for in vitro technology include cell suspension cultures, organ cultures, elicitation strategies, and bioreactor scale production.

### 4.1. Organ Cultures

Studies suggest that the approach of using various organ cultures (shoots and roots) for the production of bioactive secondary metabolites from medicinal plants is very much successful. There are many economically viable plant secondary metabolites produced from organ cultures. The metabolite of interest, Artemisinin, is also produced from organ cultures such as shoots and hairy roots. The following subsections provide details on the production of artemisinin from different organ cultures.

#### 4.1.1. Shoot Culture

When the growth of organs from an explant is administered artificially with the help of growth regulators, in synthetically prepared media, this is referred to as organ culture. It can be either a vegetative explant (root, leaf or shoot- tips) or a reproductive (flower, ovary and others) one. Specifically in the treatment of malaria, drug artemisinin and its semisynthetic derivatives have shown effects against species of *P. falciparum*. It is not only used in the treatment of malaria; the drug also finds importance as an anti-cancerous agent and is active against both chloroquine-sensitive and resistant strains of *Plasmodium* sp. Thus, the content of secondary metabolites must be elevated in the plant body by means of other compounds such as additional elicitors. Another bottleneck to this is the low level of production of artemisinin (0.01–1%) in plants, which can be improved by the production of the same using tissue culture approaches or whole plants of *A. annua*. Various branches of plant biotechnological advances have helped in the development of increased production of artemisinin in plants. One such report includes the usage of 4.4 µM BA and 0.35 µM IBA to yield the highest shoot production [14]. A good amount of variability has been observed in the case of regenerated plants with in vitro tissue culture protocols with respect to the amount of artemisinin as well as its precursors, artemisinic acid and dihydroartemisinic acid.

Keeping the issues of somatic variation and seasonal bacterial invasions on the plants in mind, ultimately leading to low organic synthesis, in vitro plant tissue culture solves the complexity of low yield to a high extent [35]. There has not only been a suspension of cells and calluses, but there has also been in vitro cultivation of shoots and roots for the production of artemisinin [36,37,38]. For the synthesis of artemisinin, there should be a minimum degree of differentiation. While multiple shoot cultures could yield trace amounts of artemisinin, it was negligible in the case of suspension cultures according to Paniego and Giulietti (1994) [24]. In contrast, a high percentage of artemisinin was found in shoots that were cultured on a ½ MS medium, along with an NAA of 0.05 mg/L, BA of 0.2 mg/L and 2% sucrose [39]. Overall, the highest amount of artemisinin was obtained from the full-bloomed flowers when they were grown with gibberellic acid in vitro [25]. The content of artemisinin was higher in shoots as compared to roots, but cultured shoots with roots showed a higher utility than those in shoots without roots [40,41]. There are several other elicitors too which can enhance the rate and concentration of artemisinin production once grown in vitro.

Scientific reports help us to understand that significant artemisinin is produced from shoot cultures of *A. annua* and the shoot cultures were established using diversified explants. Seeds of *A. annua* were used for establishing shoot cultures on an MS medium supplemented with 0.5% sucrose and 0.2 mg/L BAP along with 0.05 mg/L NAA. The same shoots, when cultured on a medium with 0.5 g/L casein hydrolysate, showed an increase in artemisinin content of 169% [39]. The MS medium supplemented with 4.4 µM BAP in combination with 0.35 µM IBA induced shoots from the seed explants and optimal multiplication of in vitro shoots is observed on media supplemented with 0.9 µM BA along with 0.05 µM NAA. It is observed that artemisinin content increased by 7.8-fold when compared to conventional seedlings [14]. Artemisinin was quantified in shoot cultures developed from leaf explants induced on an MS medium with 0.5 mg/L BAP along with 0.05 mg/L NAA. Multiplication of the shoots was successfully observed and the highest artemisinin was recorded on an MS medium supplemented with 7 mg/L GA_3_ [42].

Artemisinin content expressed in shoot cultures seems to have a correlation with the root number. A study carried out by Ferreira and Janick (1996) [43] suggests that the highest artemisinin content (0.287% in DW) was reported when shoot cultures were maintained on a hormone-free medium with the highest number of roots. When the roots were removed, artemisinin and its associated secondary metabolites were decreased almost totally by 53 to 60% [43].

#### 4.1.2. Hairy Root Culture (*Agrobacterium rhizogenes* Mediated Transformation)

Hairy root culture is an ideal tool to study secondary metabolism in plants as they exhibit a high growth rate, genetic stability and can grow in a hormone-free medium. To produce artemisinin under large-scale culture conditions, hairy root cultures were optimized but failed to give good yields. The process of hairy root formation can be initiated using *A. rhizogenes*, a Gram-negative soil bacterium that possesses the capacity to integrate its ‘Ri plasmid’ (root inducing) into the host genome after co-integration of the foreign gene inside it. Of the many reports on plasmid insertion in *A. annua* using *A. rhizogenes*, one includes a report by Ahlawat (2012), which claims that the transformants are genetically stable, rapidly growing and produce a higher level of metabolite [44]. The emergence of proliferative roots in a hairy form from the wounded area is essential from a commercial point of view when focusing on secondary metabolites of plants. It is not only of importance to the field of research and development but also to pharmaceutical industries. As a solution to the problem of endangered species in plants and decreasing quantity of secondary metabolites as a result, in vitro tissue culture protocol can always be of utility. Besides, usage of hairy root culture might help to increase secondary metabolites in plants [45]. There are a number of factors such as appropriate media, pH of media, bacterial strains and others that play a vital role in the development of hairy roots along with secondary metabolite levels in plants.

When the *A. rhizogenes* LBA 9402 strain transformed hairy root cultures of *A. annua* selected on the basis of high metabolite content, the highest productivity rose up to 390 μg/L/day when measured in terms of volume. Along with that, the combination of plant growth regulators placed and the in vitro culture media should be chosen accurately, as that determines the level of metabolite content in the regenerated plant. Among the many plant growth regulators available, 0.5 mg/L TDZ and 0.1 mg/L IAA exhibited the highest occurrence of regeneration (75%) according to Beigmohammadi et al. (2021) [46]. Besides, there needs to be an utmost focus on the type of strain being applied while transforming the plant in *A. rhizogenes*-mediated hairy root formation as that also plays a major role in the degree of transformation. When the process was attempted using four bacterial strains, A4, ATCC 15834, MSU 440, and MAFF-02-10266, the highest transformation frequency (80.7%) was observed using MSU 440, as confirmed by PCR, in addition to reflecting the highest artemisinin content (0.05%), as shown using HPLC [46].

An antimalarial drug, artemisinin, has served as a good biopharming agent too. Once genetically engineered, the metabolite can be produced at an industrial scale, thereby reducing the cost of production and increasing its availability to the public. The production and utility of genetically engineered organisms for the industrial benefits of humankind refer to biopharming technology. Thus, genetic manipulation of sources of artemisinin, *A. annua,* once carried out using *A. rhizogenes,* can elevate the amount of artemisinin content in the plants as proven by an array of experiments. Hairy root-based transformation using the above bacterium has already been used for improving the quantitative content of target products. Instead of using antibiotic-resistant genes, herbicides and the natural process of insertion of *rol* B and *C* genes into the host genomes should be favored. Another experiment was performed on similar lines along with wild-type *A. rhizogenes,* which exhibited high transformative capacity [47]. Hairy roots and calluses were produced, which in turn were producers of medicinal substances and produced a high amount of metabolite content at an accelerated rate. Further biochemical characterization and observation were carried out prior to utilizing it for biopharming research. Very interesting results by Dilshad et al. (2015) describe the higher expression of the *rolB* gene as compared to *rolC* in the formation and accumulation of artemisinin and its derivatives in the transgenics on being transformed using *A. rhizogenes* [48]. While the maximum increase of artemisinin was visible in the case of the *rolB* gene (nine-fold), the second-highest increase was credited to the *rol C* gene (four-fold). The gene responsible for the expression of the trichome index, *TAFR1,* was also expressed highly in transgenic plants as compared to wild ones. Keeping the genes responsible for increased artemisinin content in mind, transformation can be executed using a natural genetic engineer, *Agrobacterium*, thereby uplifting the overall level of artemisinin production worldwide and making it available at cheaper rates for treatment of malaria along with other biopharmaceutical applications.

### 4.2. Callus and Cell Suspension Cultures

Callus induction and the development of cell suspension culture from explants have received a lot of attention, especially for the production of bioactive molecules from medicinal plants. Calluses with parenchymatous cells, produced when tissues are cultured, formed by proliferation of the parent tissue are attributed to the callus culture, while the growth of cell aggregates in a liquid media is attributed to cell suspension culture. At the cellular level, it will be simple to investigate metabolic variations under stress or the action of any exogenous elicitor.

G.D. Brown (1993) reported that artemisinin is produced from the callus grown on an MS medium supplemented with 0.5 mg/L NAA along with 0.5 mg/L BAP, and it is seen that the biosynthetic capabilities for artemisinin production are similar to that of the mature plant [49]. Traces of artemisinin were detected in the cell suspension cultures established from the callus induced on an MS medium supplemented with 1.0 mg/L IAA along with 1.0 mg/L BAP from leaf explants. The expression of low artemisinin may be due to the high peroxidase activity [50]. Calluses developed from seeds grown on a sterile MS medium supplemented with 0.1 mg/L KN and 1.0 mg/L 2,4-dichlorophenoxy acetic acid (2,4-D) were used for the establishment of cell suspension cultures. It is seen that n-hexane and chloroform extracts showed traces of artemisinin along with some potential flavonoid compounds with anti-malarial activity against *P. falciparum* [51]. Calluses were developed on an MS medium supplemented with 0.5 mg/L of NAA and KN used for establishing cell suspension cultures with 0.1 mg/L of NAA and KN. In these cultures, the artemisinin was found to be 5.93 times less than that of the elicited precursor fed [52].

## 5. *Agrobacterium tumefaciens* Mediated Transformation for Increased Metabolite Content

Besides using plant growth regulators, plant genetic engineering helps in the production of high levels of artemisinin using recombinant DNA from the *Agrobacterium*. The transfer of a segment of its T-DNA to the host species is the most distinguishing feature of *Agrobacterium tumefaciens* that enables this soil bacterium to be of great importance in causing infection to plants. In order to increase the metabolite content in plants, root and shoot cultures of *A. annua* were transformed using *A. tumefaciens* strain T37 in a hormone-free medium. The artemisinin content found in them was 0.018 ± 0.004% dry weight (DW) [38]. In the above study, there was a higher concentration of artemisinin of around 300–400% when GA_3_ was supplemented with the media as compared to that in the control and lower levels of artemisinin (0.01% DW) in the case of transformed root cultures. There were prominent results obtained when Kiani et al. in 2012 [53] tried to increase the artemisinin production from *Artemisia dubia* using transformed plants via *A. tumefaciens* strain LBA4404 with pRT99 [53]. Interestingly, there was a two-fold increase (0.855 µg/g DW) in artemisinin concentration in transformed roots through *A. tumefaciens* as confirmed using HPLC. In another report, in which two strains of *A. tumefaciens* of nopaline wild-type were used, the artemisinin content of the transformed type (0.063 ± 0.002 g/100 g DW) was higher than in the non-transformed (0.0179 ± 0.002 g/100 g DW) one [54]. In addition, factors such as strain or explant type and age of explant were responsible for changes in tumorigenesis frequency. As a causative agent of crown gall, 2–3% of the galls produced on infection with *A. tumefaciens* that generated in vitro could regenerate spontaneously, thereby producing shooty teratoma of varied phenotype.

The expression of specific desirable genes, such as farnesyl diphosphate synthase and others, can also be initiated using transformed plants such as *A*. *tumefaciens*. It is necessary because it serves as an important precursor in the synthesis of higher metabolites such as dolichols, ubiquinones, carotenoids, sterols and others, along with being an important constituent of isoprenoid biosynthesis [55]. Using leaf discs as explants, various antibiotic-resistant shoots such as kanamycin were produced, which consisted of an optimal concentration of 20 mg/L [56]. The process includes the introduction of farnesyl diphosphate synthase gene in the cDNA and transferring it into the *A. annua* using *A*. *tumefaciens* strain LB4404. Modern technologies, such as PCR and Southern and Northern blotting helped to confirm the transformation, subsequent integration of foreign genes into the plant genome and expression of the gene at a transcriptional level, respectively. Further analysis revealed the presence of a 2–3 times higher quantity of artemisinin (8–10 mg/g DW) in the transformed plants as compared to that of the non-transformed ones. The procedure can be applied not only in the case of the farnesyl diphosphate synthase gene, but also to introduce other desirable genes into the plant system using this natural genetic engineer. When analyzing the real importance of the metabolite artemisinin, it is highly essential to bring down the cost of processing the drug and make it available for commercial purposes, as the only reliable source for it is *A. annua* (0.01–0.8% DW). Furthermore, more alternatives of production need to be researched—one of the most practical ones is development using *A. tumefaciens* from other naturally present sources. One such report includes that of Dilshad et al. (2015), in which *Artemesia carvifolia* Buch was used for genetic transformation. The metabolite content was determined by LC-MS after transformation of the wild-type shoots using *A. tumefaciens* GV3101 on inserting the *rolB* and *rolC* genes. Both genes are modulators of plant cell growth. While the former gene is related to making cells more tolerant to environmental stresses, thereby increasing the defense potential, the latter is easily connected to the growth of hairy roots. There was a remarkable 3–7-fold increase in the artemisinin content in transgenic plants with the *rolB* gene and 2.3–6-fold for the *rolC* gene as compared to the control ones. For the genes involved in the biosynthesis of artemisinin, such as cytochrome P450 and aldehyde dehydrogenase 1, differential expressions of genes were observed using real-time qPCR, which revealed a higher transcription level in the transformed ones [48].

In association, there was an enhancement in the trichome index of those transformed with the *rolB* and *rolC* genes, which is connected to the higher level of artemisinin in the same. This also demonstrates that plant secondary metabolism is affected by *rol* genes. Thus, many types of desirable genes can be introduced into the plant species using *A. tumefaciens* in order to elevate the artemisinin content and make the antimalarial drug easily available for commercialization at cheaper rates by increasing the number of sources of the metabolite.

## 6. Elicitation Strategy for Production of Artemisinin

Plant cells are considered to be biochemical factories for producing high-value secondary metabolites. There are different strategies employed to improve the yield of bioactive compounds for commercial purposes. Elicitation is one of such successful strategies for the production of secondary metabolites. The addition of biotic and abiotic components into the culture medium, with organ cultures and cell suspensions, would aid in the regulation of the metabolism of metabolite production. For artemisinin production, a conventional breeding technique was employed that resulted in new superior breeds, yielding 2% artemisinin DW. A major limitation of this method is the formation of heterozygotes that can show a huge variation in their ability to produce artemisinin. Elicitation of plant cell cultures with physical, chemical and biological agents can drastically improve the yield of metabolites from plants. Furthermore, for its best performance, one has to optimize various influential parameters such as elicitor dose, contact time, growth regulator, age and type of the explant selected and nutritional requirements of the cell [32].

### 6.1. Biotic Elicitation for Artemisinin Production

Endophytic fungi *Colletotrichum sp. B501* cell wall-derived oligosaccharide was tested for its elicitation effect on *A. annua* hairy root cultures for better yield of artemisinin. Hairy root culture in the late growth phase was exposed to elicitor at a concentration of 20 mg/L for four days, which enhanced the yield by 68.29% in comparison to the control. When examined, the peroxidase activity increased in the culture supplemented with elicitor-indicating cells, which were under stress conditions. Furthermore, cellular morphological studies revealed nuclear fragmentation and cell shrinkage leading to programmed cell death. As a part of the plant’s defense strategy, the hairy root culture reportedly produced a more valuable secondary metabolite—artemisinin [57]. Cerebroside C is a natural glycosphingolipid associated with the fungal plant pathogens *Fusarium oxysporum*, *Pythium* sps, and *Botrytis* sps and is reported to be a non-race specific elicitor for plant secondary metabolites. Studies emphasize the significant role of cerebroside as an effective elicitor in the production of artemisinin using hairy root culture. Studies have reported cerebroside to be a novel and potential lipid-based elicitor that can result in a higher accumulation of artemisinin produced by both MAV and MEP pathways. Exposure of cerebroside induced oxidative bursts in the hairy root culture, liberating nitric oxide (NO). This is one of the significant signaling molecules for the biosynthesis of artemisinin. NO inhibitors and NO scavengers when supplemented in media were able to deteriorate artemisinin production [58].

Zhang et al. (2008) [59] studied the potential role of NO in the biosynthesis of artemisinin using a hairy root culture of *A. annua* root that was elicited with a fungal-derived oligosaccharide. The oligosaccharide isolated from *Fusarium oxysporum* mycelial biomass was supplemented into the culture media which resulted in the generation of NO and an enhanced accumulation of artemisinin by 20-day-old hairy root culture, after four days of exposure to elicitor in order to confirm the participation of NO as a signaling molecule in the biosynthesis of artemisinin. The culture was treated with sodium nitroprusside (SNP) the NO donor and OE (oligosaccharide elicitor), which has resulted in a maximum yield of the metabolite recorded as 28.5 mg/L—a two-fold increase compared to the culture treated with OE alone. This suggests that generations of endogenous NO in the biosynthesis of artemisinin can trigger with the fungal-derived oligosaccharide elicitor [59].

*Colletotrichum* sp. is an endophytic fungus living in mutual association with *A. annua*. Mycelial extract of this fungus was proved to be a potential elicitor of the hairy root culture of *A. annua*. The 23-day-old late-phase culture was exposed to 0.4 mg total sugar ml^−1^ of the elicitor for four days, showing a 44% enhancement of artemisinin accumulation. There was an increase in metabolite production with an increase in dose concentration of the elicitor [60]. *P. indica,* as an endophytic fungus, can colonize on the root and penetrates the root system with its hyphae, further proliferating in the root cortex. Fungi can induce the root cells to produce a precursor for artemisinin production such as artemisinic acid and dihydroartemisinin in roots. These are translocated to shoot where they are transformed into artemisinin [61]. An embryogenic callus obtained from the leaf disc of *A. annua* was induced to produce regenerated plantlets on MS media with 5.0 mg/L 6-BA and 1.0 mg/L NAA and incubated at 25 ± 1 °C under 16 h photoperiod. Both calluses and callus-regenerated plantlets were exposed to endophytic fungi *P. oxalicum* B4 to elicit the cultures for artemisinin production. Calluses showed a negative response with browning of tissue, necrosis and no artemisinin, while rooted plantlets after 30 days of exposure to endophytic fungi resulted in a better yield of artemisinin, which was estimated to be 1.32 mg/g DW, about 43.5% more than the control [62]. *A. annua* shoots derived from nodal explants were cultured on different media such as MS basal media, MS media + 5 μM IBA, MS + *P. indica* and MS + 5 μM IBA + *P. indica*. Root regeneration was less in media without *P. indica*. Artemisinin content was significantly high in shoots established on MS media with 5 μM IBA + *P. indica* [61].

### 6.2. Abiotic Elicitation for Artemisinin Production

Nano elicitors are gaining more importance in secondary metabolite production using plant cell culture as they can alter physiological activities and biochemical processes inducing the plant cells to make secondary metabolites. Ag-SiO_2_ NPs were used as an elicitor for hairy root culture for a better yield of artemisinin. NPs sized 101.8 ± 8.9 nm were supplemented into culture media at a concentration of 900 mg/L for three days. This has induced oxidative stress in a hairy root culture due to the gradual release of Ag^+^ resulting in malonyldialdehyde (MDA), an indicator of lipid oxidation, along with a high generation of hydrogen peroxide and catalase activity. Artemisinin production was estimated to be 13.3 mg/L, which was 3.9-fold better in yield compared to the control [63]. Cell suspension cultures of *A. annua* were subjected to chitosan nanoparticles of varying concentrations (5–15 mg/L) that were examined for their effect on artemisinin production and expression levels of genes associated with the biosynthesis of artemisinin after 8, 24, 48 and 72 h of treatment. Studies confirmed that 5.0 mg/L was more effective in elicitation with improved yields of artemisinin and upregulated expression of ADS, CYP, CPR, DBR2 and ALDH genes [64]. A callus derived from the leaf disc of *A. annua* on MS media with 0.5 mg/L NAA, 0.5 mg/L BAP, and 30 g/L sucrose was elicited with cobalt nanoparticles for better artemisinin production. A total of 5.0 mg/L cobalt nanoparticles supplemented into media enhanced artemisinin production after 24 h of elicitation, while its concentration decreased with time. To understand the effect of nanoparticle elicitation on the expression of two key genes involved in biosynthesis of artemisinin, SQS and DBR2, it was analyzed using qRT-PCR. These genes had a negative influence on artemisinin production. Expressions of these genes were low when the callus was elicited with cobalt nanoparticles. On the other hand, HPLC quantification reported an increase in artemisinin yields. The increase in the artemisinin content can be attributed to the activation of other genes involved in the biosynthetic pathway. Furthermore, to have a very clear understanding of the elicited product, it is essential to check the expression of various genes involved in the biosynthesis of the product rather than just studying two or three genes of the biosynthetic pathway [65].

A low artemisinin-producing cell suspension culture of *A. annua*, sp. was subjected to elicitors, such as ultraviolet B (UV-B) and dimethyl sulfoxide (DMSO), to improve the yield, but elicitation had no effect on the yield of artemisinin. PCR studies revealed the absence of ADS, DBR2 and ALDH1, whose products have a significant role in the biosynthesis of artemisinin [66]. DMSO was used as an abiotic elicitor for in vitro regenerated *A. annua* shoots with roots. DMSO elicitation was performed on unrooted shoots which did not show any improvement in artemisinin production compared to the rooted shoot that was stimulated by the elicitor. This emphasizes the significant role of the root system in artemisinin production in shoots. Furthermore, DMSO elicitation resulted in the liberation of ROS, which was studied with DAB staining. The rooted plantlets showed an increased DAB stain, indicating ROS is used in shoots as a defense strategy, while unrooted plantlets failed to show any ROS in their leaves. The study has shown that the expression of two gene ADS (amorphadiene synthase) and CRP (amorpha-4,11-diene monooxygenase) involved in the biosynthesis was monitored using RT-PCR. The ADS transcript did not show any response to DMSO elicitation, while CYP transcript was inversely proportional to the artemisinin produced [67].

Native β-cyclodextrins were chemically modified to heptakis (2,6-di-O-methyl)-β-cyclodextrin (DIMEB), which was used as a chemical elicitor in cell suspension culture of *A. annua* for artemisinin production. The effect on DIMEB and methyl jasmonate was studied separately towards metabolite production. Studies confirmed the combined effect as 50 mM DIMEB and 100 μM methyl jasmonate were optimal with the highest artemisinin of 27 μmol/g dry weight which was 300-fold higher than untreated suspension culture [68]. Oligogalacturonides are damage-associated molecular patterns (DAMPs) released by wounded and injured plant cells. This can serve as a potent elicitor in plants stimulating early defense mechanisms, inducing the plants to produce ROS, which in turn induces plants to produce secondary metabolites [69]. Oligogalacturonides (OGA) are readily obtained from plant tissues using pectinase enzyme, which was further purified using column chromatography. This OGA with polymerization DP = 4.57 when supplemented into a hairy root culture media at a concentration of 60 g/mL for four days increased the yield of artemisinin production to 11.3 mg/L which was 55.2% more than the control without the elicitor [70].

Gamma radiation is an efficient abiotic elicitor that has enhanced the yield of artemisinin in the callus culture of *A. annua*. Gamma radiation of different doses (5–35 Gy) was used to study its effect on callus growth, cell survival and artemisinin production. The growth and regeneration of calluses are reduced with increasing doses of gamma radiation. To examine its impact on artemisinin production in five successive in vitro subcultures, artemisinin was quantified using HPLC. A low dose of gamma radiation was found to be effective as an elicitor for artemisinin production when compared to a non-irradiated callus culture. This confirms that a low dose of gamma radiation resulted in mutants with enhanced artemisinin production [71]. A low-yielding artemisinin-producing chemotype of the *A. annua* variety (136 P) was subjected to elicitation with UV-B radiation for different time intervals and varying concentrations of DMSO to stimulate the suspension culture and soil-grown 136 P. There was no elicitation or stimulation in artemisinin production in the case of suspension culture while there was enhanced yield of artemisinin in soil-grown plants [66].

Cell suspension culture of *A. annua* was set to study the effect of sodium acetate (SA), mevalonic acid lactone (MAL), casein acid hydrolysate (CAH) and cholesterol (CH) as precursors in artemisinin production. A positive response with a better yield of artemisinin was recorded with sodium acetate (25 mg/L), mevalonic acid lactone (50 mg/L) and casein acid hydrolysate (500 mg/L). Feeding the media with sodium acetate resulted in increased acetylco-A in the cell which can boost cellular activities for secondary metabolite production. When the culture was supplemented with mevalonic acid lactone (MAL), it served as the precursor of the lactone ring that enhanced the production of artemisinin. Supplementing casein acid hydrolysate into media will provide aromatic amino acids that can improve the yield of artemisinin. When abiotic elicitor methyl jasmonate was supplemented along with a mevalonic acid lactone (MAL) precursor, it proved to be the best combination as this has resulted in a synergetic effect, with a maximum yield of 110.20 mg/L, which was 5.93 times more than the control [52].

GA_3_ had a positive effect on the shoot culture of *A. annua* for artemisinin when supplemented at a concentration of 10 mg/L in MS media. A significant increase in artemisinin was observed at the blooming stage and its effect was only for one cycle, but its effect on artemisinin production ceased in later cycles. In another approach, sterol biosynthesis inhibitors (miconazole, naftifine and terbinafine) were used so that the C15 intermediates are channeled towards artemisinin production. Among the examined inhibitors, naftifine showed an excellent result with its increase in concentration of the product obtained, while terbinafine was toxic to culture and minconazole had a negative impact [39].

This is a study of the synergetic effect of two elicitors on artemisinin production using the cell suspension culture of *A. annua*. Cells when treated with varying concentrations of sorbitol resulted in osmotic stress in plant cells, which was monitored by estimation of MDA and H_2_O_2_ that are considered to be the indicators of oxidative stress. When the culture was pre-treated with coronatine (Cor), it greatly reduced this oxidative stress, as it can stabilize the cell membrane, maintain the integrity of plant cells and also stimulate antioxidant activities to overcome stress. Cell cultures showed a better yield of artemisinin when exposed to 30 g/L of sorbitol. Beyond this, the concentration had a negative impact on artemisinin production due to the ROS-mediated cell disruption. Pre-treatment of cell cultures with Cor also resulted in increasing the yield of artemisinin, but the combined effect drastically increased the yield eight-fold [72].

Shoots generated from the sterilized seeds of *A. annua* on MS media containing 30 g/L sucrose and 10 g/L agar were maintained at 25 ± 2 °C for 16 h under cool-white light (35 mmol m^−2^s^−1^). These shoots were transferred to liquid MS media and subjected to elicitation with silver and copper nanoparticles. Studies confirmed that when nanoparticles were used at concentrations above 10 μM, they inhibited the shoot growth and reduced chlorophyll content. These heavy metal NPs induced oxidative stress liberating more H_2_O_2_, which augmented the production of secondary metabolites such as artemisinin as a defense strategy in shoots [73]. The hairy root culture of *A. anuua* was optimized for increased artemisinin production with gibberellic acid which had a profound effect on its growth. Artemisinin production increases as gibberellic acid induces branching of roots, induction of flowering and accumulation of artemisinin will be always high before flowering [36].

When the culture was elicited with methyl jasmonate (MeJA) and fungal elicitors (*Alternaria alternate*, *Curvularia limata*, *Fusarium solani*, and *Piriformospora indica*), farnesyl pyrophosphate and miconazole, the results obtained indicated the synergistic effect of abiotic (MeJA at 100 µM) and biotic (CH of *P. indica* at 3% *v*/*v*), elicitors improving the production of artemisinin [74]. The *A. annua* hairy root culture was subjected to chitosan, methyl jasmonate and yeast extract as the elicitors to stimulate the culture for a better yield of commercially important artemisinin production. Studies confirmed that all these elicitors when supplemented in MS media at concentrations of 200 µM (methyl jasmonate), 2.0 mg/L (Yeast extract) and 150 mg/L (chitosan) were found to be the optimal dose of elicitors for the highest yield of bioactive substances, recorded as 1.52 ± 0.32 and 0.95 ± 0.01 mg/mg dry wt, and 1.84 ± 0.02 mg/mg dry wt., respectively [75].

In order to know the effect and to optimize the concentration of elicitors on biomass and artemisinin production using a hairy root culture of *A. annua*, RSM studies were performed with 20 experiments designed with three elicitors—namely casein acid hydrolysate, sodium acetate and methyl jasmonate. The steep increase in the yield of artemisinin was recorded as 3.45 mg/g when cultured in MS media with methyl jasmonate (40 µg/L), casein acid hydrolysate (50 µg/L) and sodium acetate (500 µg/L), while in the control, the yield was only 0.4 mg/g [76]. The hairy root culture of *A. dubia* was transformed with *A. rhizogenes* LBA9402 that was elicited with gibberellic acid and salicylic acid for artemisinin production. Gibberellic acid exhibited a better elicitation potential at a low concentration of 0.01 mg/L with 80 ± 3 µg/g of DW, which was 93% more than the control. Salicylic acid could also stimulate the culture at a concentration of 13.8 mg/L yielding 79 ± 3 µg/g of artemisinin, which was 38% more than the control [77].

A callus that was initiated from the leaf explant of *A. absinthium* was cultured on MS media using the raft culture method to investigate the influence of growth factors and amino acids. This culture, when maintained with cysteine amino acid at a concentration of 12.5 mg/L, resulted in 2.8 µg/g of artemisinin, while when media were supplemented with growth factors such as BAP (2 mg/L) and NAA (2 mg/L), it improved the yield of artemisinin to 3.05 µg/g and 1.95 µg/g, respectively. Other combinations of amino acids and growth factors had the least effect on the culture regarding artemisinin production [78]. Table 3 depicts the data of comprehensive research on elicitation of cell and organ cultures using biotic and abiotic agents for artemisinin production.

## 7. Bioreactor Scale Production of Artemisinin

Bioreactors are considered to be the heart of industrial biotechnology aiming at the mass production of commercial and pharmaceutical products for humankind. Construction and optimization of the various factors for optimal functioning of the bioreactor are challenging fields of research. The main factors considered in designing a plant bioreactor are multiplication rate, oxygen, and nutrient transfer, reduction in power supply, labor and space. These are generally automated machines that facilitate the controlled operation for a better yield of biomass and metabolites. However, there are limitations that can reduce the performance of bioreactors. The needs to be addressed are the accumulation of inhibitors, foaming, shear stress, contamination and hyperhydricity. By eliminating these limitations, bioreactors can be the ideal tool for mass production of metabolites in a short period of time at a large scale to satisfy the global demand [79].

The work by Patra et al. (2016) [80] focused on improving the growth and artemisinin production from hairy root culture with three different designs of bioreactors, namely bubble column, nutrient mist and modified nutrient mist bioreactors, which were operated in batch mode. Switching from a stirred tank bioreactor to a bubble column bioreactor showed better growth of the hairy root culture when all these bioreactor efficiencies for artemisinin production were evaluated based on the yield of artemisinin and biomass. Among the bioreactor tested, Modified NMB was better in both biomass and artemisinin yield followed by NMB and the column bioreactor. The main reason for better yield with Modified NMB is due to effective oxygen and nutrient supply. In total, 1.12 mg/g of artemisinin with biomass of 23.03 g/L was reported with Modified NMB followed by 0.22 mg/g of artemisinin and 8.52 g/L of biomass with NMB, and the least was with BCR, reporting 0.27 mg/g of artemisinin and biomass of 5.68 g/L [80]. Liu et al. (2003) [81] used a 25-day batch culture of shoots obtained from *A. annua* to optimize the yield of artemisinin for commercial applications. In order to examine the efficiency of the modified airlift bioreactor, a multi-plate radius-flow bioreactor and an ultrasonic nutrient mist bioreactor shoot culture were initiated and maintained in these bioreactors. The modified airlift bioreactor resulted in the lowest yield of artemisinin as shoots were hyperhydrated leading to vitrification. The mist bioreactor provided excellent support compared to the multiple plate radius bioreactor. Nutrients were provided in the gas phase as a mist, which ensures a good nutrient supply to cultures that improved the yield of artemisinin by 3.3- and 1.4-fold, compared to the airlift and multiple plate radius reactor, respectively. The amount of artemisinin obtained was recorded as 48.2 mg/L after 25 days in the mist bioreactor [81]. The MNB (mist nutrient bioreactor) was found to be the most potential bioreactor for the mass production of artemisinin. Further operating conditions were standardized for improving the yield. A 25-day batch culture was initiated with a shoot culture of *A. annua* in MNB to ensure homogenous growth of the shoots in the bioreactor. The mist cycle was optimized to 3/90, in which the mist was on for 3 min and off for 90 min, which also resulted in the highest biomass and artemisinin yield, which were 13.4 and 46.9 mg/L [82].

Wyslouzil et al. (2001) [83] standardized the key operating parameters for the highest artemisinin production, using hairy root cultures of *A. annua* in MNB. Three key factors, namely mist cycle, carrier gas and nutrient composition, that can influence the kinetics of the root and the accumulation of artemisinin, were considered in this experiment. Setting the mist cycle can directly influence the root growth as well as the artemisinin production, as they can distribute the nutrients uniformly in the bioreactor and also mediate oxygen transfer. A 1 min on/15 min off mist cycle resulted in a good branching of the root with a fresh weight of 4.6 ± 1.0 mg, while 5 min on/15 min off caused browning of the root and necrosis with a fresh weight of 3.4 ± 1.4 mg. A 1 min on/60 min off mist cycle resulted in thinner roots with the least branching of roots with a fresh weight of 3.9 ± 0.6 mg. Furthermore, when 1% CO_2_ was used as the carrier gas, it had no impact on root growth but greatly reduced necrosis. Conditioned B5 media were found to be more effective in maintaining the roots in the log phase for a long time, and branching was also extensive [83]. Patra and Srivastava (2015) [84] used mathematical models to standardize the feed batch culture for the highest yield of artemisinin using a modified stirred tank bioreactor. Sucrose is a significant carbon source in plant tissue culture media and an osmoregulator that can greatly influence the in vitro propagation of roots in a bioreactor. The fed-batch culture can easily overcome the nutrient limiting factor for improving the yield of secondary metabolites. The application of the model design of the fed-batch culture can improve its performance. A modified Monod’s model was employed to optimize the addition of sucrose to the fed-batch culture of the hairy root culture of *A. annua* for the highest yield of artemisinin. Sucrose (37 g/L) was fed at a constant rate of 0.1 L/day for 10–15 days, which resulted in improved artemisinin accumulation of 0.77 mg/g, while another batch culture, which was maintained in a pseudo-steady state fed with (20.8 g/L) for 10–15 days, resulted in the highest artemisinin accumulation recorded as 0.99 mg/g [84].

Patra and Srivastava (2014) [85] set a batch culture of a hairy root from *A. annua* to examine and optimize the factors associated with the operation of a stirred tank bioreactor for the high accumulation of artemisinin in the culture. When the culture was set beyond 70 rpm, it resulted in sheer stress. Similarly, other parameters, such as temperature (25 °C), size of inoculum (1 g/L DW), age of inoculum (8 d) and medium to vessel volume ratio (0.18), were optimized for the best performance of the stirred tank bioreactor towards the mass production of artemisinin. A high biomass accumulation of 6.3 g/L dry weight (37.50 g fresh weight) and 0.32 mg/g was obtained after an incubation period of 25 days [85]. A batch culture was initiated in an internal loop bioreactor with an ultrasonic mist cycle for improved growth and artemisinin production from the adventurous roots of *A. annua*. The mist cycle was set in such a way that every 90 min interval, three mins of misting was applied for effective nutrient supply and oxygen transfer. After 25 days of incubation, with an airflow rate of 0.5 L/min, artemisinin yield was estimated as 46.9 mg DW/L of the culture media [86]. Kim et al. (2001) confirmed that the hairy root culture could yield three times more artemisinin in a nutrient mist bioreactor (NMB) than in a bubble bioreactor [87]. The hairy root culture is one of the recommended cultures for the mass production of plant secondary metabolites due to its genetic stability, good productivity and ease in genetic manipulations. In these studies, bubble bioreactors and modified bubble bioreactors were used to analyze the growth and artemisinin production of the hairy root culture. After 25 days of incubation, improved artemisinin was produced and a modified bubble bioreactor was observed [88]. Table 4 depicts the data of comprehensive research on bioreactors and culture conditions for the production of artemisinin.

## 8. Metabolic Engineering for Artemisinin Production

Conventional and in vitro modes of artemisinin production in plant systems alone can not meet the demand for artemisinin, as biosynthetic studies revealed that their production is restricted to specific tissues such as glandular trichomes. From recent studies, it is known that modern biotechnological applications such as metabolic engineering have helped in deriving the required metabolites from plants and microbes. It can be seen that terpenoids were produced by engineering mevalonate pathways in *E. coli*. Studies carried out by Martin et al. (2003) showed that the yield of precursors such as amorpha-diene was increased by engineering the amorpha-4,11-diene synthase gene from *Saccharomyces cerevisiae* [89]. Similarly, there are many studies carried out on plant model systems that helped in the enhancement of artemisinin. Previously, human therapeutic proteins were successfully produced in plant model systems, such as tobacco, using metabolic engineering techniques. The success rate was significant [90,91].

The expression of amorpha-4,11-diene is particularly significant in metabolic engineering for the manufacture of artemisinin and it is produced from the precursor farnesyl diphosphate. Wallaart et al. (2001) [92] successfully cloned the cDNA sequence for the expression of amorpha-4,11-diene synthase in *E. coli* and they could reach 50% identity. Later, upon introduction to the tobacco plant system, they found that it resulted in significant expression. This transgenic tobacco model system successfully helped in the production of amorpha-4,11-diene (0.2–1.7 ng/g FW) [92]. Amorpha-4,11-diene is expressed more than 1000-fold along with some other novel terpene molecules when cytosolic carbons and plastid’s isoprenoids are overexpressed and diverted among their compartments. Co-expressing plastid-targeted ADS and FPS helped in increasing the yield of amorpha-4,11-diene (25 µg/g FW) in tobacco [93]. Employing synthetic biology, Farhi et al. (2011) tried to create a mega vector with cytochrome P450, ADS, CYP71AV1, DBR2 and a de-regulated tHMGR gene from yeast. In spite of being strategically robust at the molecular level, the yield of artemisinin is comparatively lower (around 7 µg/g DW) [33]. Agroinfiltration of the constructs with amorpha-4,11-diene synthase, 3-hydroxy-3-methylglutaryl-CoA reductase and FPS regions helped in the expression of amorpha-4,11-diene in leaves of *Nicotiana benthamiana* [94].

## 9. Conclusions and Future Prospects

Extensive research on artemisinin indicates that it is an effective anti-malarial drug with a variety of other pharmacological properties. This review reveals that the traditional method of producing artemisinin in vitro and from glandular trichomes is insufficient, and that specific biotechnological techniques are essential. Hairy root technology along with elicitation of cell and organ cultures of *Artemisia* species will help to reach the expectation in terms of the production of artemisinin. When compared to conventional methods of production, bioreactor-scale production of artemisinin might be regarded as a substantial alternative. The characterization of other species of Artemisia can be explored for the quantification of artemisinin, and simultaneous tissue culture studies can be explored for artemisinin production. Biotechnological applications, such as metabolic engineering in other *Artemisia* species and heterogeneous host technology, can yield better results, allowing for more exploration for the sustainable production of artemisinin.

## Figures and Tables

**Table 1:** In vitro regeneration of *Artemisia annua* through direct organogenesis from various explants.

Explant	Media	Response	References
Seed	MS + 0.1 ppm BA + 1.0 ppm NAA	Shooting	[12]
Inflorescence	MS + 1.0 mg/L BAP + 2.0 mg/L IBA	Multiple shooting	[23]
Stem	MS + 0.1 mg/L TDZ	Multiple shooting	[15]
Leaf, petiole	MS + 1.0 mg/L TDZ	Shooting and rooting	[20]
Seed	MS + 0.1 mg/L KN + 0.01 mg/L NAA	Shooting	[13]
Nodal stem explants	MS + 0.2 mg/L NAA + 0.2 mg/L BAP	Multiple shooting	[16]
Nodal stem explants	Shooting: MS + 4.44 µM BAP Rooting: ½ MS + 2.46 µM IBA	Multiple shooting and rooting	[17]
Nodal stem explants	Shooting: MS + 10.0 µM 2-iP; Rooting: 1/2 MS + 5.0 µM NAA	Multiple shooting and rooting	[18]
Nodal stem explants	Shooting: MS + 0.8 mg/L BAP + 0.1 mg/L IBA; Multiplication medium: MS + 1.0 mg/L BAP + 0.1 mg/L IBA; Rooting: 1/2 MS + 0.5 mg/L IBA	Multiple shooting and rooting	[19]
Leaf	Shooting: MS + 1.0 mg/L BAP + 0.05 mg/L NAA + 2.0 mg/L AgNO_3_	Shoot regeneration	[21]
Leaf	Shooting: MS + 0.5 mg/L NAA + 2.0 mg/L BA; Rooting: MS + 0.1 mg/L IBA	Shooting and rooting	[22]
Seed	Shooting: MS + 4.4 µM BA + 0.35 µM IBA; Multiplication medium: MS + 0.9 µM BA + 0.05 µM NAA	Shooting and multiplication	[14]

**Table 2:** In vitro regeneration of *Artemisia annua* through indirect organogenesis from various explants.

Explant	Media	Response	References
Leaf, hypocotyl	Callus: MS + 5.4 µM NAA; Shooting: MS + 13.32 µM BA + 1.08 µM NAA	Callusing and organogenesis	[24]
Hypocotyl	Callusing and shooting: MS + 0.5 µM NAA + 13 µM BAP + 0.3 µM GA_3_	Callusing and multiple shooting	[25]
Leaf	Callusing: MS + 0.1 mg/L BAP + 0.05 mg/L NAA; Shooting: 0.4 mg/L BAP + 0.2 mg/L NAA	Callusing and multiple shooting	[16]
Leaf	Callusing: MS + 1.0 mg/L BAP + 0.05 mg/L NAA	Callusing and organogenesis	[26]
Leaf	Callusing: MS + 0.5 mg/L NAA or 2,4-D + 0.5 mg/L BAP; Shooting: 0.25 mg/L NAA + 1.0 mg/L BAP; Rooting: ½ MS + 0.1 mg/L IBA	Callusing and organogenesis	[27]

**Table 3:** Various biotic and abiotic elicitors and culture conditions used for the production of artemisinin.

Elicitor	Culture Type	Culture Conditions	Yield of Artemisinin	Reference
**Biotic elicitors**				
Cell wall’s oligosaccharide from *Colletotrichum* sp. B501	Hairy root culture	MS medium + 20 mg/L elicitor	Increased by 68.29%	[57]
Cerebroside from fungal source	Hairy root culture	MS medium + 10–70 µg/mL cerebroside	Increased by 2.3 folds	[58]
Oligosaccharide from *Fusarium oxysporum* mycelium	Hairy root culture	MS medium + 0.3 mg total sugar/mL elicitor	Increased from 0.7 mg/g DW to 1.3 mg/g DW	[59]
Mycelial extract of *Colletotrichum* sp.	Hairy root culture	MS medium + 0.4 mg total sugar/mL elicitor	Increased from 0.8 mg/g DW to 1 mg/g DW	[60]
*Pencillium oxalium* B4	In vitro grown Rooted plantlets	MS medium + 5.0 mg/L BAP + 1.0 mg/L NAA + *P. oxalium* B4 (30 days exposure)	Increased by 43.5%	[62]
**Abiotic elicitors**				
Ag-SiO_2_ nanoparticles	Hairy root culture	MS medium + 900 mg/L nano elicitor	Increased by 3.9 folds	[63]
Chitosan nanoparticles	Cell suspension culture	MS medium + 0.5 mg/l NAA + 0.5 mg/L BAP + 15 mg/L Elicitor	NA	[64]
Cobalt nano particles	Callus culture	MS medium + 0.5 mg/L NAA + 0.5 mg/L BAP + 5 mg/L elicitor	Increased by 2.25 folds	[65]
Chitosan	Hairy root culture	MS medium + 150 mg/L chitosan	Increased by 6 folds	[75]
Oligogalacturonides	Hairy root culture	MS medium + 0.01 mg/L gibberellic acid +60 g/mL elicitor	Increased by 55.2%	[70]
Heptakis (2,6-di-O-methyl)-β-cyclodextrin (DIMEB) and methyl jasmonate	Cell suspension culture	MS medium + 2.0 mg/L 2,4-D + 0.15 mg/L BAP + 50 mM DIMEB + 100 µM Methyl jasmonate	Increased by 300 folds (27 umol/g DW)	[68]
Methyl jasmonate and mevalonic acid lactone	Cell suspension culture	MS medium + 0.1 mg/L NAA + 0.1 mg/L KN + 50 mg/L mevalonic acid lactone + methyl jasmonate	Increased by 5.93 times	[52]
Sorbitol and Coronatine	Cell suspension culture	MS medium + 0.1 mg/L NAA + 0.1 mg/L KN + 30 g/L Sorbitol + 0.05 µM Coronatine	Increased by 8 folds	[72]

**Table 4:** Bioreactors and culture conditions for the production of artemisinin.

Culture Type	Type of Bioreactor	Culture Conditions	Yield	References
Hairy root culture	Modified nutrient mist bioreactor	¼ MS + 10 µg/L GA_3_Batch culture having adequate oxygen supply and nutrient	1.12 mg/g	[80]
Shoot culture	Mist bioreactor	MS + 0.05 mg/L NAA + 0.5 mg/L BAP; 25 days batch	48.2 mg/L	[81]
Shoot culture	Mist nutrient bioreactor	MS + 0.05 mg/L NAA + 0.5 mg/L BAP; 25 days batch	46.9 mg/L	[82]
Hairy root culture	Mist nutrient bioreactor	B5 medium; 1 min on/15 min off mist cycle	NA	[83]
Hairy root culture	Modified stirred tank bioreactor	¼ MS + 10 µg/L GA_3_; Fed batch for 10–15 days	0.99 mg/g DW	[84]
Hairy root culture	Stirred tank bioreactor	MS medium; 25-day batch.	0.32 mg/g DW	[85]
Adventitious roots	Mist bioreactor	MS medium; 0.5 L/min air, 25-day culture	46.9 mg DW/L	[86]

## Data Availability

Not applicable.
